# Provincial-level outcomes of China’s ‘Reducing maternal mortality and eliminating neonatal tetanus’ program

**DOI:** 10.1038/s41598-020-70257-x

**Published:** 2020-08-07

**Authors:** Peiran Chen, Mingrong Li, Jun Zhu, Yanping Wang, Yi Mu, Qi Li, Li Dai, Zheng Liu, Juan Liang, Hanming Liu

**Affiliations:** 1 National Office for Maternal and Child Health Surveillance of China, West China Second University Hospital, Sichuan University, Chengdu, Sichuan China; 2 Key Laboratory of Birth Defects and Related Diseases of Women and Children (Sichuan University), Ministry of Education, Ren Min South Road Section 3 No. 17, Chengdu, Sichuan China; 3 Department of Obstetrics, West China Second University Hospital, Sichuan University, Chengdu, Sichuan China; 4 Department of Pediatrics, West China Second University Hospital, Sichuan University, Ren Min South Road Section 3 No. 17, Chengdu, Sichuan China

**Keywords:** Health care, Health policy, Health services, Public health

## Abstract

To determine whether the nationwide program ‘Reducing maternal mortality and eliminating neonatal tetanus’ contributed to the rapid decline in China’s maternal mortality ratio (MMR) and neonatal tetanus elimination by enhancing hospital delivery, we compared MMR and neonatal tetanus incidence rate (NTR) reductions by province from 2000 to 2013. The difference-in-difference method was used to analyze the program effect. Long-term effects were analyzed relative to MMR and NTR in 2000 and 2002, respectively, while short-term effects in a given year were analyzed relative to MMR and NTR in the preceding year. The national program was associated with a faster decline in MMR in the long term. The rate of decline showed an inverse ‘U’ shape from 2000 to 2013, peaking in 2009. The program had a short-term effect in MMR reduction in 2005, 2007, and 2009. The program was also associated with faster decline in NTR in the short term at some time points, but this association was not consistent and was not found in the long term. In conclusion, the program accelerated decline of MMR from 2000 to 2013 but did not clearly reduce NTR at the province level. Therefore, this targeted program worked efficiently in resource-poor areas.

## Introduction

Reducing maternal mortality is challenging. It requires comprehensive changes in policy support, health financing, health workforce, health infrastructure and maternal care coverage^1^. Unfortunately, the global 2015 goal for the reduction in maternal mortality was not met. A World Health Organization (WHO) report released at the end of 2015 showed that the global maternal mortality ratio (MMR) fell by 44% from 385 to 216 deaths per 100,000 live births between 1990 and 2015, which fell short of the 75% decline stipulated in Millennium Development Goal 5 (MDG 5)^2^. In addition, no region of the world achieved that. Reducing maternal mortality in resource-scarce areas such as Africa and remote areas of China is even more difficult.

Annual MMR reduction rate in China was only 2.8% from 1996 to 1999^3^, which is less than the 5.5% that WHO estimated to be needed in order to achieve MDG 5. One of the challenges to reduce maternal death in China is the regional inequity in healthcare infrastructure and access. For example, MMRs are nearly three times higher in western areas than eastern areas^4^, and the annual MMR reduction in western areas was only half that in eastern areas^3^. Estimates suggest that more than 95% of maternal deaths in western areas and 76% in eastern areas are preventable, with obstetric hemorrhage accounting for 59% of preventable maternal deaths in western regions and 35% in eastern regions^5^.

In addition to reducing maternal mortality, the Chinese government also aimed to reduce neonatal death. The Plan for China's children's development in the 1990s, which was released in 1992, stated that China aimed to decrease MMR by 50%, decrease neonatal mortality by 33% by 2000, and eliminate neonatal tetanus by 1995^6^. However, a study of 30 counties from six provinces in China found that the average neonatal mortality rate was 22‰ in 1996. Of the neonatal deaths, 16.3% were attributed to neonatal tetanus, and this was still the third most frequent cause of neonatal death in 1996. In total, 89% of tetanus-related deaths involved births at homes, where family members assisted^7^. These deaths would have been preventable if the births had occurred with skilled attendants under disinfected and sanitary conditions.

In order to reduce preventable maternal and neonatal deaths, the Chinese government launched the nationwide program ‘Reducing maternal mortality and eliminating neonatal tetanus’ in 2000. The goal of this program was to reduce the national MMR and neonatal tetanus incidence rate (NTR) in remote, poor areas in the central and western parts of the country. The program aimed to increase hospital births and provide skilled attendants and disinfectant birth environment. It involved eliminating the ‘four inaccessibilities’: (1) eliminate the culture inaccessibility, by providing health education to maternal and child health (MCH) workers and women of reproductive age and their families. Health education aims to persuade women to give birth in a hospital. It includes information on maternal health, the benefits of hospital delivery, and possible risks of traditional delivery; (2) eliminate economic inaccessibility by providing financial assistance to poor families for hospital births; (3) eliminate territory inaccessibility by providing maternal emergency centers, referral networks and ‘green channels’ for first aid; and (4) eliminate service inaccessibility, by training grass roots clinicians or sending them to provincial hospitals for further training. Training was mainly provided for midwifery skills, including manual removal of the placenta, repair of vaginal lacerations, cesarean section, and first aid skills. Besides, the program also provide basic and emergency obstetric equipment, blood supplies and medicine to village, township and county medical hospitals to improve the quality and accessibility of obstetric services^8^.

It is likely that this nationwide program helped reduce MMR and NTR in China; however, most studies focused on county-level outcomes and did not examine later stages of the program^8–11^. Therefore the present study examined the program’s outcomes at provincial level in both the short and long terms.

## Methods

### The ‘Reducing maternal mortality and eliminating neonatal tetanus’ program

**Figure 1:**
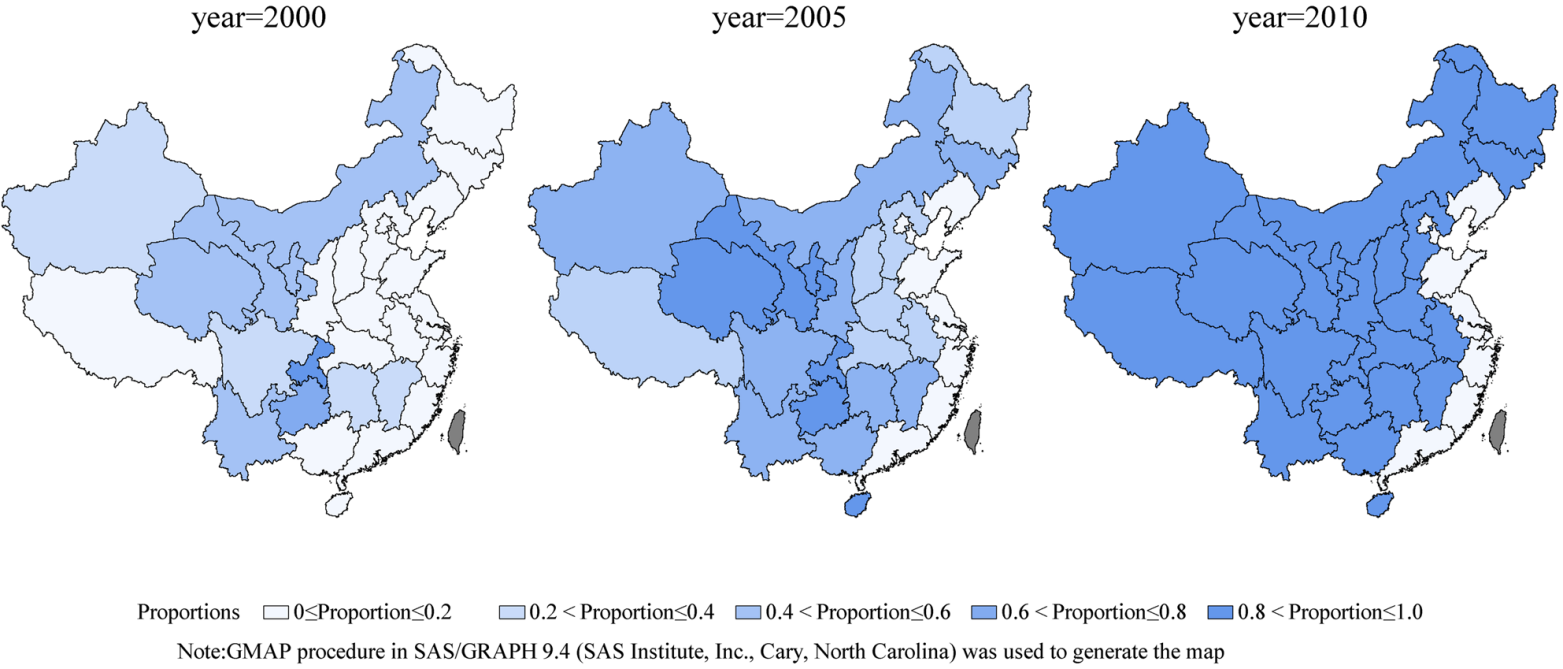
County coverage, by province, of the national ‘Reducing maternal mortality and eliminating neonatal tetanus’ program in mainland China in 2000, 2005 and 2010. Shading reflects the proportion of counties in each province that were included in the program.

This government-initiated program was started in 2000 in 378 counties across 12 provinces where MMRs were > 80 per 100,000 live births (Inner Mongolia, Jiangxi, Hunan, Chongqing, Sichuan, Guizhou, Yunnan, Tibet, Gansu, Qinghai, Ningxia and Xinjiang). In 2004, the program was expanded to include another 62 counties in Jilin, Hubei, Guangxi, Shaanxi and Xinjiang provinces and was expanded again in 2005 to another 560 counties from Hebei, Shanxi, Heilongjiang, Anhui, Henan, Hainan and Xinjiang provinces. Except for resource-rich provinces along the east coast, the program included all provinces by 2005. In 2008, 182 counties were added to the program. The program included all counties in the enrolled provinces for a total of 2,288 counties from 22 provinces by 2010. Starting in 2014, the program was integrated into the National Essential Public Health Programs (NEPHPs), which subsidized hospital births to ensure long-term effects. Figure 1 shows the program expansions in mainland China.

### Data

Data on provincial MMRs were collected through China’s Provincial MCH Surveillance system (PMCHSS) from 2000 to 2013. No data were available for 2001. MMR was calculated as ${\text{MMR}} = \frac{{{\text{Maternal }}\;{\text{deaths}}\;{ }({\text{direct }}\;{\text{cause }}\;{\text{and }}\;{\text{indirect}}\;{\text{ cause}})}}{{{\text{Live}}\;{\text{ births}}}} \times 100,000$. Maternal deaths were defined as pregnant women who died during pregnancy or within 42 days of pregnancy termination, regardless of pregnancy duration or site. Maternal deaths due to accidental or incidental causes unrelated to pregnancy or its management were not considered. Live births were defined as newborns alive after at least 28 weeks of pregnancy, or a live newborn weighing more than 1 kg according to the definition of perinatal birth in China^12^.

The PMCHSS is a five-level network that involves villages, townships, counties, municipalities, and provinces. Data on the numbers of live births and deaths in women of reproductive age in the village are initially collected by village doctors who are responsible for health management. The village doctors then report the data to township MCH workers. The process for data collection is as follows: data are collected from township MCH institutions by provincial MCH institutions level by level. The accuracy of number of live births and maternal deaths, as well as authenticity of cause of maternal death, are checked before being reported to higher levels. Most provinces have electronic reporting systems and collect data online. The provincial MCH institution finally report the maternal death and live birth data to the National Office for MCH Surveillance every year using a standardized form.

Cases of maternal death and deaths in women of reproductive age are required to be reported to township MCH institutions within 24 h by village doctors. The deceased woman’s family members are visited by an obstetrician or MCH worker from the township hospital within three days to inquire about the cause of death and to determine if the woman was pregnant. If the woman was pregnant, relevant information is reported to MCH institutions at county level. MCH staff at county then conduct a further, more comprehensive investigation that includes collecting information from family members, midwife, anesthetist, fellow physicians and nursing staff of the deceased woman through standardized forms, and by obtaining a copy of the medical records. Standardized forms are completed within seven days and submitted to the commission at county level.

All maternal deaths are reviewed by the relevant commission at county, municipal, and provincial levels. After reviewing the deaths, the commission first determines whether they met the criteria for a maternal death and then identifies the main cause of death, factors that contributed to the death, and preventive measures that were subsequently implemented. The death review results are submitted to the National Office for MCH Surveillance, which reviews the data to rectify possible errors^3^.

To ensure the quality of collected data, all the data go through a quality control process each year. The quality control process, which aims to investigate missing reports and data quality, is multisource and multilevel. All the data and information for deaths of woman in reproductive age from hospitals, Centers for Disease Control (CDC), police and crematoriums are collected and cross-checked. After excluding reported maternal deaths and deaths of women who were not pregnant, the remaining possible maternal death cases are investigated by inquiring the hospital or local community to determine the cause of death. If deaths was confirmed as pregnant woman, a supplement or missing case report is initiated. The quality control procedures occur from bottom to top, starting at the county level, then progressing to municipality, province, and national levels. Quality control procedures occur once per year at provincial and national levels, four to six times per year at county levels, and twice per year at all other levels. There is a permitted threshold of < 15% missing reports for maternal deaths and < 10% for live births. If the reported data fails to meet the requirements during the quality control procedure at each level, MCH institutions reinvestigate the missing report and resubmit reports to meet requirements. When quality control occurs at each level, the missing report rate is approximately 5%, which is far below the permitted threshold.

**Figure 2:**
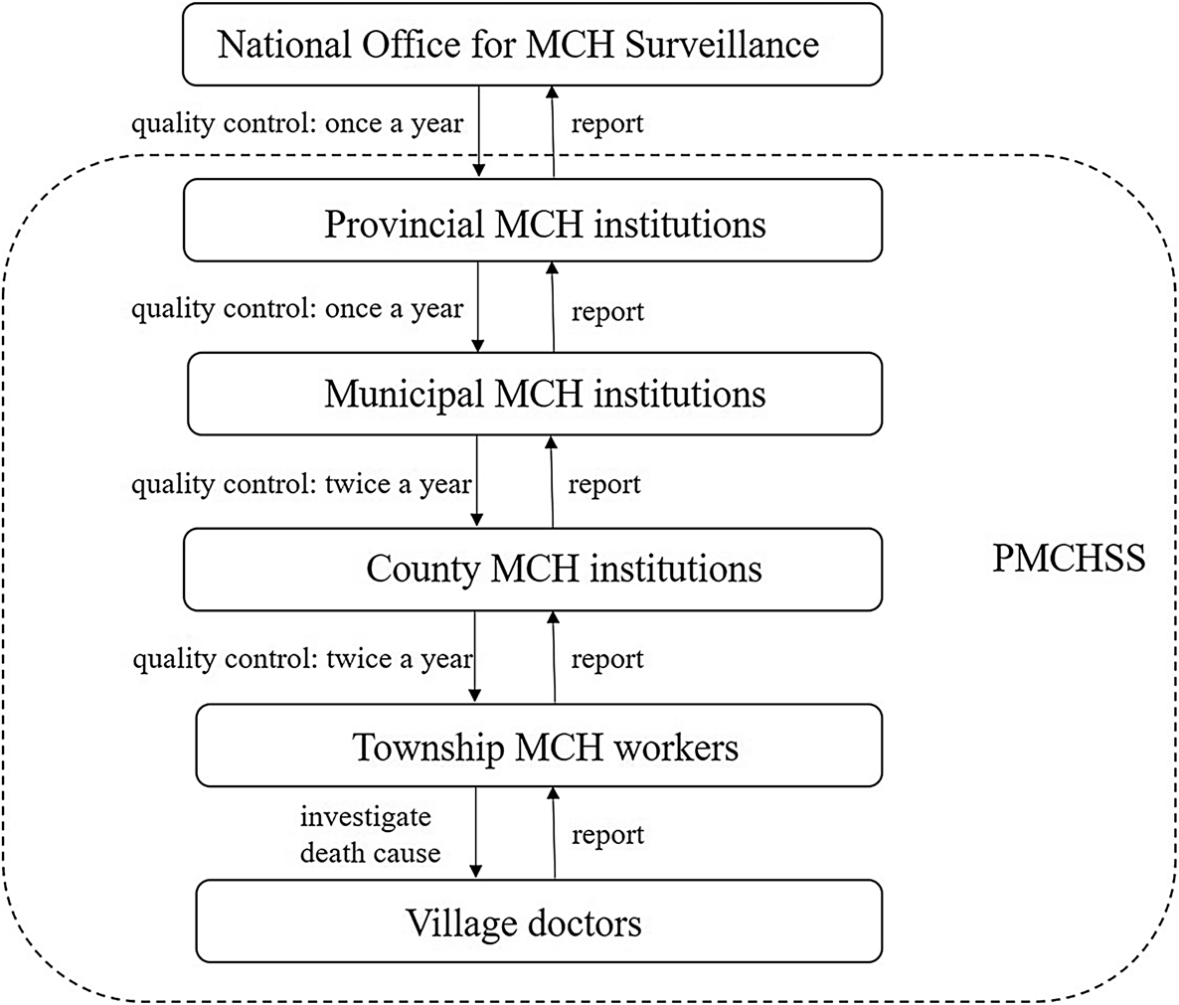
Data reporting and quality control procedures.

The data collection and quality control details are shown in Fig. 2.

NTR data at provincial level was collected through the yearbook of National Health Statistics from 2002 to 2013. Data were not available for 2000 or 2001. NTR data at county levels from 2000 to 2013 were collected from the ‘Reducing and Eliminating’ program and quality control measures information as described above for PMCHSS.

Data on the following covariates were collected through the yearbook of China Statistics from 2000 to 2013: licensed physicians per 1,000 people, illiteracy rate, regional GDP (Gross Domestic Product), and miles of highways per 1,000 miles.

Neonatal tetanus elimination was a binary variable and was defined as less than one neonatal tetanus case per 1,000 live births at district level, in accord with WHO criteria^13^.

### Statistical analysis

The program’s effects were analyzed for the long-term effects on reducing maternal death and neonatal tetanus (henceforth termed the ‘long-term analysis’), the short-term effect on reducing maternal death and neonatal tetanus (‘short-term analysis’), and the trend in neonatal tetanus elimination among counties in the program (‘elimination analysis’).

Provinces outside the program were used as the control. In the long-term analysis, MMR in 2000 and NTR in 2002 were taken as the baseline. In the short-term analysis, MMR and NTR in the year before the year being analyzed were used as the baseline. The difference-in-difference method (DID) was used for the long- and short-term analyses. This method uses data from before and after the intervention and controls to assess the program’s effects while avoiding selection bias^14,15^. To control for other potential confounders, analyses were conducted with the following covariates: licensed physicians per 1,000 people, illiteracy rate, regional GDP and miles of highways per 1,000 miles.

The model used for long- and short-term analysis can be summarized as.

$$ U_{it} = \beta_{0} + \beta_{{{\text{post}}}} *{\text{Post}} + \beta_{\exp } * {\text{Exposure}} + \beta_{{{\text{interaction}}}} *{\text{Post}}*{\text{Exposure}} + \beta_{1} *{\rm X}_{it} + \varepsilon_{it} , $$

where *U*_*it*_ is the MMR or NTR of province *i* at time *t*, *β*_post_ and *β*_exp_ are binary variables indicating whether the province *I* was in the postinclusion period or was or was not enrolled in the program at time *t*, the interaction is for the DID estimates, and *X*_*it*_ captures the covariation of province *i* with time *t*.

Provinces were categorized into four groups according to whether they were never included in the program (group g0) or were included in 2000 (g1), 2004 (g2) or 2005 (g3). Box plots were used to illustrate the dispersion of MMR and change in MMR over time.

Logistic regression was used in the elimination analysis to examine neonatal tetanus elimination. A binary variable of neonatal tetanus elimination at county level served as the dependent variable in the model; year, as the independent variable; and hospital birth rate, as the covariate. The Mantel–Haenszel test was used to analyze neonatal tetanus elimination trends from 2000 to 2013.

We also analyzed how much individual counties participating in the program contributed to provincial MMR and NTR reduction. The model of panel data analysis was *y*_*it*_ = *α*_*i*_∗ + *β*_*i*_*x*_*it*_ + *u*_*it*_, where MMR or NTR at county level was the only independent variable, MMR or NTR at provincial level was the dependent variable, and all other variables were assumed to be uncorrelated and invariant through different time and units. The county contribution was estimated using *R*^2^^16^.

SAS 9.4 (SAS Institute, Cary, NC, USA) was used for data analysis, and significance was defined at the 0.05 level.

## Results

### Program effects on maternal death

Table 1 shows estimates of long- and short-term effects of the national program on MMR reduction. After adjustment for covariance, the program showed long-term reduction in MMR beginning in 2003, with a maximal effect in 2009 (*β* = − 54.03, *P* = 0.0036). In the short-term analysis, the program significantly reduced MMR in 2005 (*β* = − 14.58, *P* = 0.0041), 2007 (*β* = − 8.53, *P* = 0.0174) and 2009 (*β* = − 5.68, *P* = 0.0106).

**Table 1:** Coefficients (p-value) for estimating effects of the national program on MMR and NTR.

	2000	2002	2003	2004	2005	2006	2007	2008	2009	2010	2011	2012	2013
**MMR**													
Long-term effect													
	Reference	− 18.23 (0.0666)	− 21.85 (0.0375)*	− 25.47 (0.0122)*	− 42.17 (0.0015)*	− 43.80 (0.0080)*	− 46.83 (0.0055)*	− 52.49 (0.0041)*	− 54.03 (0.0036)*	− 43.91 (0.0163)*	− 38.04 (0.0064)*	− 38.79 (0.0043)*	− 41.02 (0.0032)*
Short-term effect													
	Reference	− 18.23 (0.0666)	− 3.37 (0.2948)	0.72 (0.8558)	− 14.58 (0.0041)*	− 2.53 (0.5187)	− 8.53 (0.0174)*	− 6.23 (0.1076)	− 5.68 (0.0106)*	1.65 (0.6092)	− 3.48 (0.0890)	− 3.22 (0.1403)	− 1.50 (0.3726)
**NTR**													
Long-term effect													
		Reference	− 0.02 (0.9597)	− 0.28 (0.4667)	− 0.49 (0.3989)	− 0.92 (0.2539)	− 1.13 (0.2211)	− 1.44 (0.1822)	− 1.59 (0.1649)	− 1.45 (0.2278)	− 0.40 (0.6565)	− 0.31 (0.7162)	− 0.34 (0.7047)
Short-term effect													
		Reference	− 0.02 (0.9597)	− 0.17 (0.6402)	− 0.27 (0.2940)	− 0.35 (0.0872)	− 0.11 (0.4528)	− 0.06 (0.6052)	− 0.26 (0.0140)*	0.11 (0.2354)	− 0.03 (0.5145)	− 0.06 (0.2926)	0.08 (0.2659)

Values in ‘()’ are *P*-values for each coefficient. *Program effect was statistically significant.

Estimates for program effect were adjusted for licensed physicians per 1,000 people, illiteracy rate, regional GDP and miles of highways per 1,000 miles.

**Figure 3:**
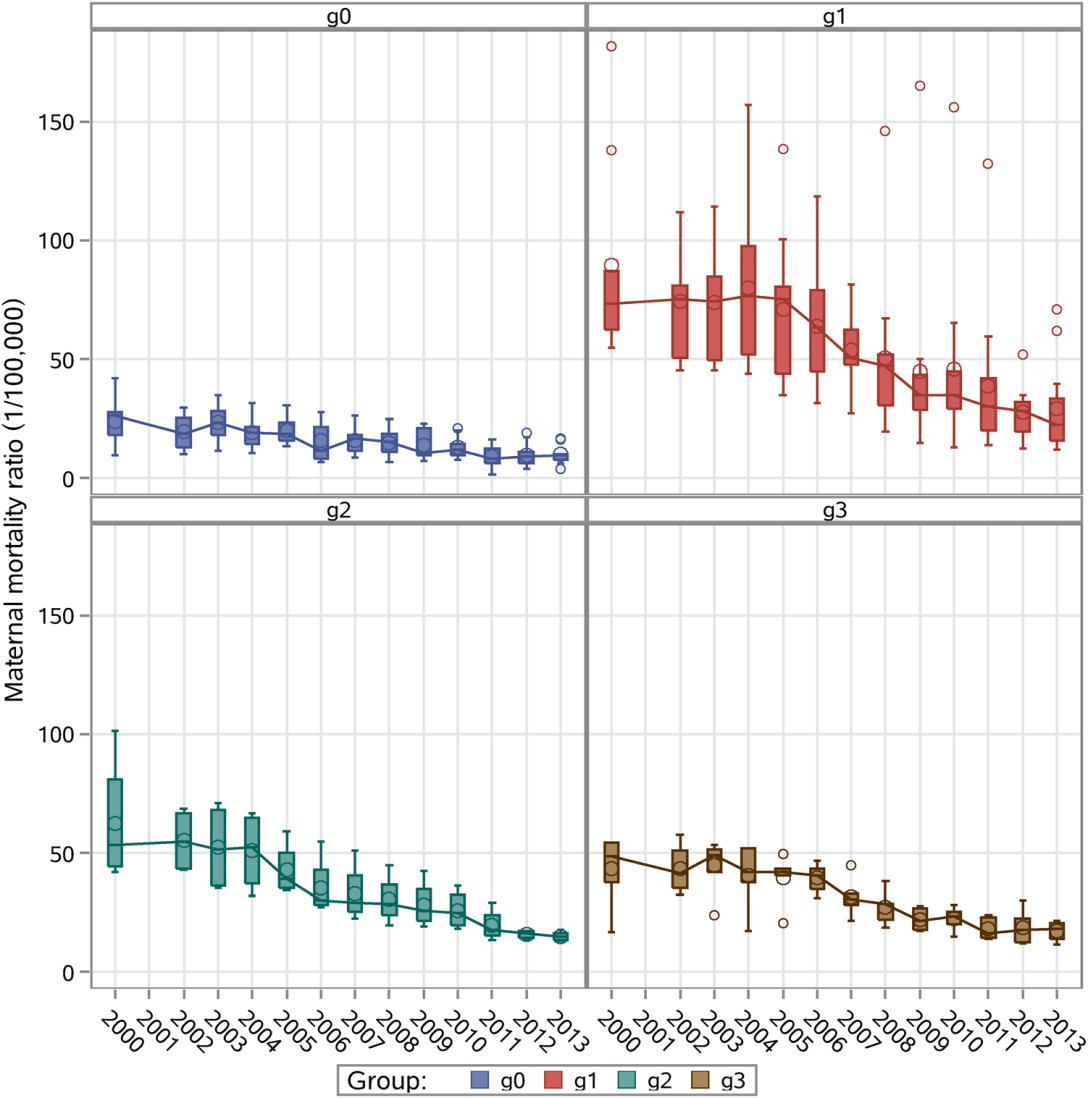
MMR during the study period for different groups of provinces. g0 = provinces outside the program; g1 = provinces in the program from 2000; g2 = provinces in the program from 2004; g3 = provinces in the program from 2005; *MMR* maternal mortality ratio.

Figure 3 shows the box plot for MMR per year in g0, g1, g2, and g3 provinces. MMR in g0 provinces remained low, with slight fluctuations. MMR in g1, g2, and g3 provinces was initially higher than in g0 provinces, but declined thereafter, closing the gap between g0 and other provinces.

**Figure 4:**
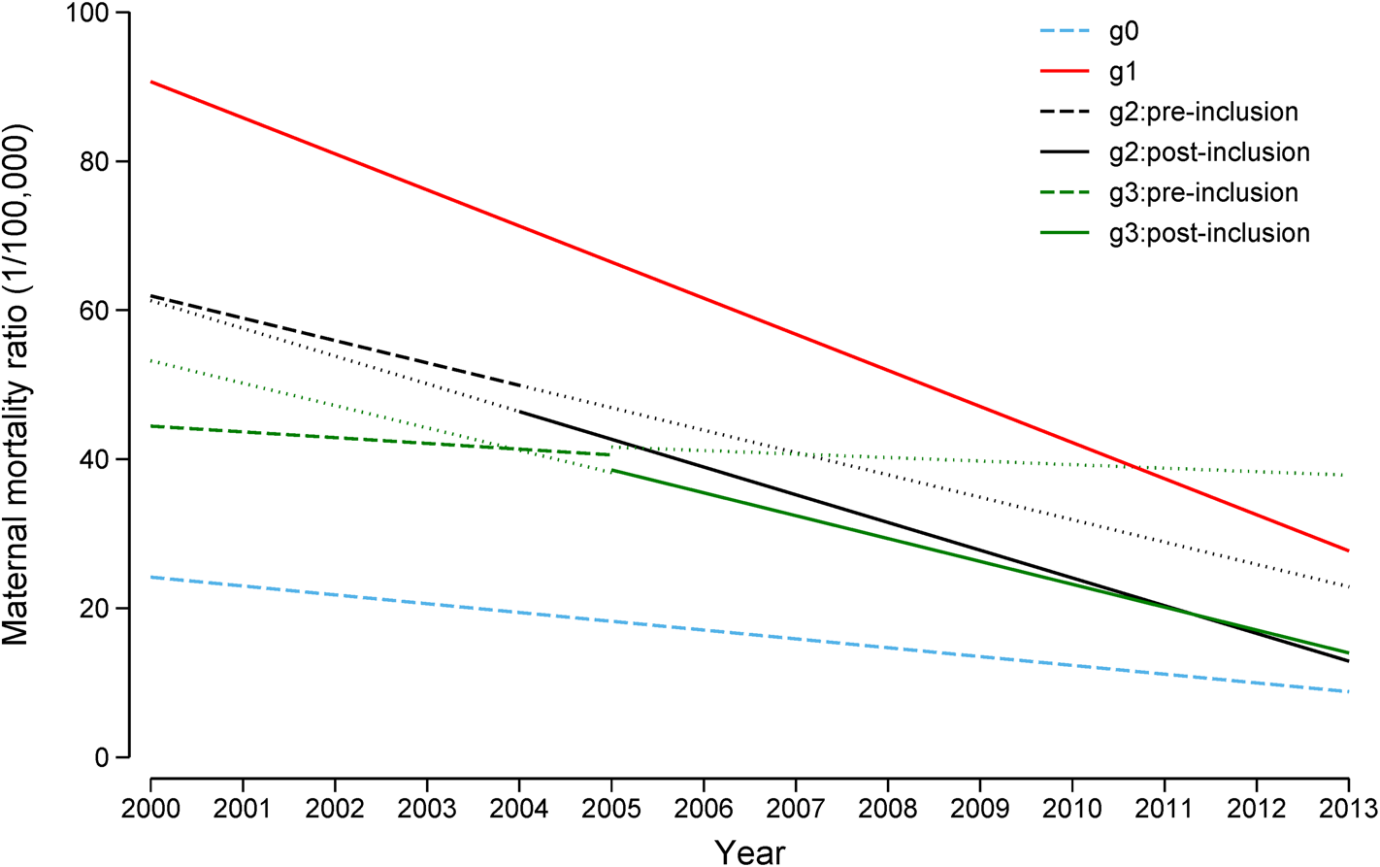
Trend for MMR in different groups of provinces. g0 = provinces outside the program; g1 = provinces in the program from 2000; g2 = provinces in the program from 2004; g3 = provinces in the program from 2005; data for provinces outside the program are shown as dashed lines; data for provinces in the program, as solid lines. *MMR* maternal mortality ratio.

The annual data in Fig. 3 are replotted as trend lines in Fig. 4. This figure shows that MMR quickly decreased in the four province categories. The g0 provinces showed the least negative slope, while g1 showed the most negative slope. Slopes for g2 and g3 provinces became more negative after their inclusion in the program.

### Program effects on neonatal tetanus

Table 1 shows long- and short-term effects of the program on NTR reduction. No significant results were observed in the long term, whereas the program reduced the NTR in 2009 in the short-term analysis (*β* = − 0.26, *P* = 0.0140).

Table 2 shows that the proportion of counties that eliminated neonatal tetanus increased from 206 (55.2%) in 2000 to 1815 (97.4%) in 2013, and shows that this trend was statistically significant (*Z* = − 39.76, *P* < 0.0001). The rate of hospital births increased from 55.2% in 2000 to 99.2% in 2013 in counties included in the program. This increasing rate was associated with a significant increase in neonatal tetanus elimination at county level (OR 6.34, 95% CI 5.05–7.95).

**Table 2:** Neonatal tetanus elimination among counties in the national program.

Year	Rate of hospital births (%)	Neonatal tetanus elimination		
		Number of counties (missing)	No	Yes
2000	55.21	373 (5)	167 (44.77%)	206 (55.23%)
2001	58.86	373 (5)	121 (32.44%)	252 (67.56%)
2002	62.37	374 (4)	110 (29.41%)	264 (70.59%)
2003	64.99	374 (4)	104 (27.81%)	270 (72.19%)
2004	70.06	424 (4)	109 (25.71%)	315 (74.29%)
2005	75.53	966 (4)	212 (21.95%)	754 (78.05%)
2006	81.10	966 (4)	171 (17.70%)	795 (82.30%)
2007	86.82	967 (3)	136 (14.08%)	830 (85.92%)
2008	90.69	1,158 (0)	124 (10.72%)	1,033 (89.28%)
2009	95.37	2,138 (4)	133 (6.22%)	2,004 (93.78%)
2010	97.14	2,003 (139)	101 (5.04%)	1,902 (94.96%)
2011	98.36	2,115 (27)	76 (3.59%)	2,039 (96.41%)
2012	98.83	2,137 (5)	67 (3.14%)	2,070 (96.86%)
2013	99.22	1,864 (278)	48 (2.58%)	1,816 (97.42%)

**Figure 5:**
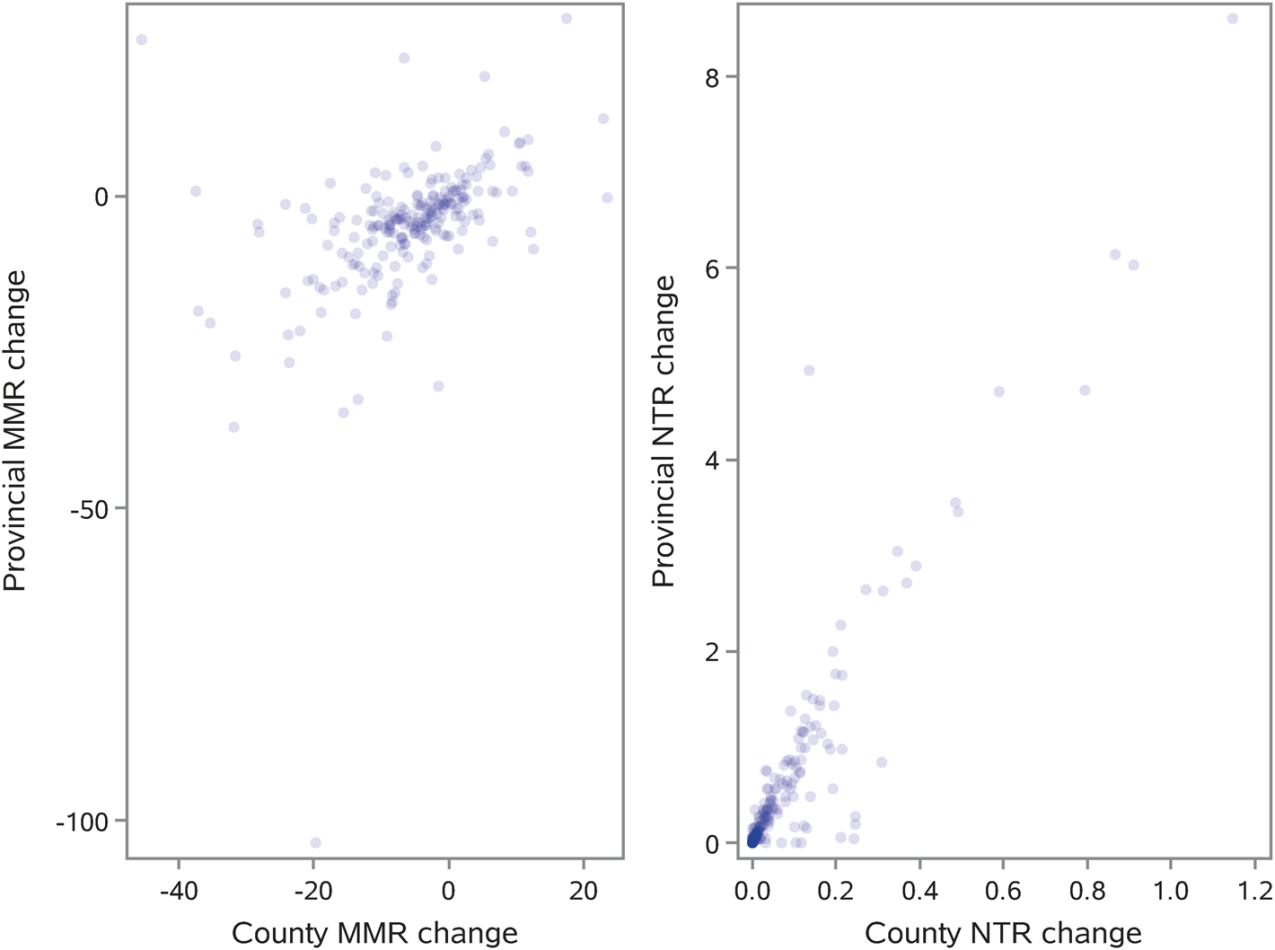
Contributions of individual counties to provincial decline in MMR and NTR. Left panel: MMR; right panel: NTR. *MMR* maternal mortality ratio, *NTR* neonatal tetanus incidence rate.

Figure 5 shows how much individual counties contributed to reducing provincial MMR and NTR. The positive trend for both outcomes was significant, with respective *R*^2^ values of 0.1569 (Fig. 5, left panel) and 0.2549 (Fig. 5, right panel).

### Details of ethics approval

All surveillance data were collected through China’s national routine surveillance systems and the study was conducted using aggregated data as part of routine surveillance activities. No formal ethical approval was required.

## Discussion

Our study provides a comprehensive assessment of provincial-level outcomes of the ‘Reducing and Eliminating’ program. Although the program was only implemented in rural counties, the results of our study show that it has accelerated MMR reduction at provincial level. Previous assessments of the program also concluded that it led to a faster MMR decline for counties included in the program compared to counties not included in the program^8,10^. However, these previous studies did not examine data from 2008 onwards. Therefore, the present study extends the program assessment through its end in 2013, when the program's measures were integrated into the NEPHPs. This study shows that programs can accelerate provincial MMRs reduction and that they require a long time to show evidence of success. Therefore, these programs can help narrow the gap in maternal and child health infrastructure between remote and coastal provinces in China (Fig. 3).

To understand the program effects in detail, we examined long- and short-term effects separately. In the long term, MMR fell faster in provinces included in the program compared with those not included in the program (Fig. 4), and the gap between the two sets of provinces increased with time before 2010 (Table 1). One explanation for this trend may be that the provincial governments, on the basis of the accelerated MMR reduction observed in provinces within the program, also began to invest in areas outside the program^17,18^ thus helping to increase the apparent effects of the program at provincial level. We performed a provincial-level analysis to capture such ‘spillover’ effects. However, this lifetime program assessment revealed that while the program accelerated MMR reduction from 2003 to 2009, continued reductions were not seen after 2009. The inverse ‘U’ shape of the program effect may have occurred because home births without hygienic standards and medical assistance were the main cause of maternal death in poor, remote areas of China when the program began. However, as the initial problem of access to adequate obstetric services was addressed by increasingly widespread hospital birth, which narrowed the gap between provinces that were in or outside the program. The program did not have statistically significant effects in 2002. This may reflect the fact that only a small proportion of counties had implemented the program in each province by 2002 since the program only began in 2000.

In contrast to the successful long-term analysis, short-term showed few significant reductions in MMR at provincial level. These results suggest that such programs need a relatively long time to show effects.

In contrast to its long-term success with MMR reduction, the ‘Reducing and Eliminating’ program did not appear to have significantly accelerated an overall decline of NTR in China. Neonatal tetanus is mainly caused by unsanitary home births and can therefore be eliminated if newborns are born in a hospital with skilled attendants and in a disinfected and sanitary environment. NTR data were available between 2002 and 2013. The program was initially launched in 2000. In the third year of the program (2002), NTR had already fallen to low levels (NTR in 2002, 1.201‰). In addition, the NTR may be relatively high in some counties, but low at provincial level. While NTR varied largely between provinces (coefficient of variation, CV = 1.78 in 2002), the program aimed to eliminated the NT at county level, Therefore, it is even harder to achieve statistically significant results at provincial level. These findings were confirmed by another study that compared the NTR between program counties and counties outside the program and showed that the program significantly reduced NTR in the early years of the program^10^. Nevertheless, the proportion of counties that eliminated neonatal tetanus increased significantly over time, and the NTR decrease at county level helped China lower its provincial-level NTR by 30% (Fig. 5, right panel).The incidence of neonatal tetanus has historically been high in China^19^ due to the poor economic situation and preference for home or traditional births^20^. Our results suggest that the increasing rate of hospital births can eliminate neonatal tetanus. A sampling study by WHO in two high-risk areas included in the program in 2014 failed to find any case of NT, leading the organization to declare its elimination from China^21^.

China’s success in reducing MMR may reflect its decision to focus efforts on disadvantaged areas from the beginning. Nepal, like China, implemented a targeted program to address high maternal and neonatal mortality^22,23^, but their MMR reduction from 1990 to 2015 was smaller than China’s^24^. Although both countries provided birthing facilities and offered economic incentives for mothers, Nepal did not initially target disadvantaged areas. In contrast, China initially introduced the program to impoverished counties with the highest MMRs in 2000, then expanded the program in 2004 and 2005. This suggests that interventions aimed at the most vulnerable areas can have impressive effects^25^.

Our study is one of the first to separately assess the national program on its long and short term effect separately. In addition, we also assessed the program effect over its whole lifespan from 2000 to 2013. However, there were still limitations to this study, including the lack of NTR data from counties that were not in the program and the lack of baseline data from provinces before their inclusion in the program. In addition, by failing to take into account potentially relevant variables such as institutional first aid capability and first aid equipment quality, our analysis may overestimate the contribution of each county to provincial MMR and NTR reduction^16^.

The ‘Reducing maternal mortality and eliminating neonatal tetanus’ program is one of the largest programs in maternal and child health that the Chinese government has funded (approximately 4 billion USD), and is also one of the longest with a lifespan of 13 years. The cumulative effect of the program and sustainment hospital births subsidies (the program was integrated into NEPHPs and expanded its coverage of subsidized hospital births to ensure long-term effects) resulted in a current hospital delivery rate over 99% and a steadily decreasing MMR in China. China achieved MDG5 in 2014^26^ and the MMR in 2019 is 17.8 per 100,000 live births^27^. Available evidence suggests that the program has paid off for MMR reduction not only in China but in all of East Asia. This is because China accounts for roughly 85% of the population in East Asia, and the great reduction in MMR of China contributed to the reduction in the entire region, which had the second largest MMR reduction in the world^2^. The program has supported maternal and child health in impoverished areas in China through health education, training, infrastructure and financial incentives. In addition, it has helped change attitudes about giving birth in a hospital, where mothers have access to hygienic conditions and medical assistance when necessary. These experiences may be useful for other countries, especially those in resource-poor areas that struggle with reducing maternal death and improving perinatal safety.

## References

[1] Gao Y (2017). Progress and challenges in maternal health in western China: a Countdown to 2015 national case study. Lancet Glob. Health.

[2] 2WHO. Health in 2015: From MDGs to SDGs. https://www.who.int/gho/publications/mdgs-sdgs/en/ (2015).

[3] Liang J (2010). Maternal mortality in China, 1996–2005. Int. J. Gynecol. Obstet..

[4] Guo Y, Bai J, Na H (2015). The history of China's maternal and child health care development. Semin. Fetal Neonat. Med..

[5] Liang J (2011). Preventable maternal mortality: geographic/rural-urban differences and associated factors from the population-based Maternal Mortality Surveillance System in China. BMC Public Health.

[6] The Ministry of Health implements the Outline of the Development Plan for Chinese Children in the 1990s. *Maternal and Child Health Care of China* **7**, 5 (1992).

[7] Ding Hui PY, Huang X, Wu L, Xiong Z, Wu M, Qi Q (1999). Epidemiological analysis on neonatal tetanus in thirty counties of six provinces in China. Chin. J. Vaccines Immun..

[8] Liang J (2012). The changes in maternal mortality in 1000 counties in mid-Western China by a government-initiated intervention. PLoS ONE.

[9] You H, Bogg L, De Costa A, Dong H (2014). Rural maternal mortality ratio in China. Lancet Glob. Health.

[10] Feng XL (2010). An impact evaluation of the Safe Motherhood Program in China. Health Econ.

[11] Liang J, Zhu J, Wang YP, Li MR (2007). Analysis on factors affecting maternal mortality in China. Zhonghua Liu Xing Bing Xue Za Zhi.

[12] WHO. *World Health Organization International Statistical Classification of Diseases and Related Health Problems *(*10th version*) (1998).

[13] WHO. *WHO-recommended surveillance standard of neonatal tetanus*. https://www.who.int/immunization/monitoring_surveillance/burden/vpd/surveillance_type/active/NT_Standards/en/ (2010).

[14] Cataife G, Pagano MB (2017). Difference in difference: simple tool, accurate results, causal effects. Transfusion.

[15] Khandker, S. R., Koolwall, G. B. & Samad, H. A. *Hand book on Impact Evaluation*, Vol. 5, 71–86 (2010)

[16] Hsiao C, Ely JC, Andrews DWK (2014). Simple Regression with Variable Intercepts.

[17] Lin F (2009). Effect analysis of the "Reducing and Eliminating" program in Qingzhen county. Matern. Child Health Care China.

[18] 18Li, L. The process and effect of the "Reducing and Eliminating" program in Anhui province. *Chin. Rural Health Serv. Admin.*, 869–871 (2008).

[19] Chai F, Prevots DR, Wang X, Birmingham M, Zhang R (2004). Neonatal tetanus incidence in China, 1996–2001, and risk factors for neonatal tetanus, Guangxi Province, China. Int. J. Epidemiol..

[20] Ministry of Health, People's Republic of China. https://data.cnki.net/yearbook/Single/N2009100139 (2003).

[21] Fan Z (2014). Validation on the elimination of neonatal tetanus programs through Lot Quality Assurance-Cluster Sample Survey in China. Zhonghua Liu Xing Bing Xue Za Zhi.

[22] Paudel M, Javanparast S, Dasvarma G, Newman L (2019). A critical account of the policy context shaping perinatal survival in Nepal: policy tension of socio-cultural versus a medical approach. BMC Health Serv. Res..

[23] Barker CE, Bird CE, Pradhan A, Shakya G (2007). Support to the Safe Motherhood Programme in Nepal: an integrated approach. Reprod. Health Matters.

[24] Ahmed SM (2016). Cross-country analysis of strategies for achieving progress towards global goals for women's and children's health. Bull. World Health Organ..

[25] Shetty AK (2016). Global maternal, newborn, and child health: successes, challenges, and opportunities. Pediatr. Clin. N. Am..

[26] Liang J (2019). Maternal mortality ratios in 2852 Chinese counties, 1996–2015, and achievement of Millennium Development Goal 5 in China: a subnational analysis of the Global Burden of Disease Study 2016. Lancet.

[27] National Health Commission of the People's Republic of China. Statistical Bulletin on China's Health Development (2019). http://www.nhc.gov.cn/guihuaxxs/s10748/202006/ebfe31f24cc145b198dd730603ec4442.shtml (2020).

